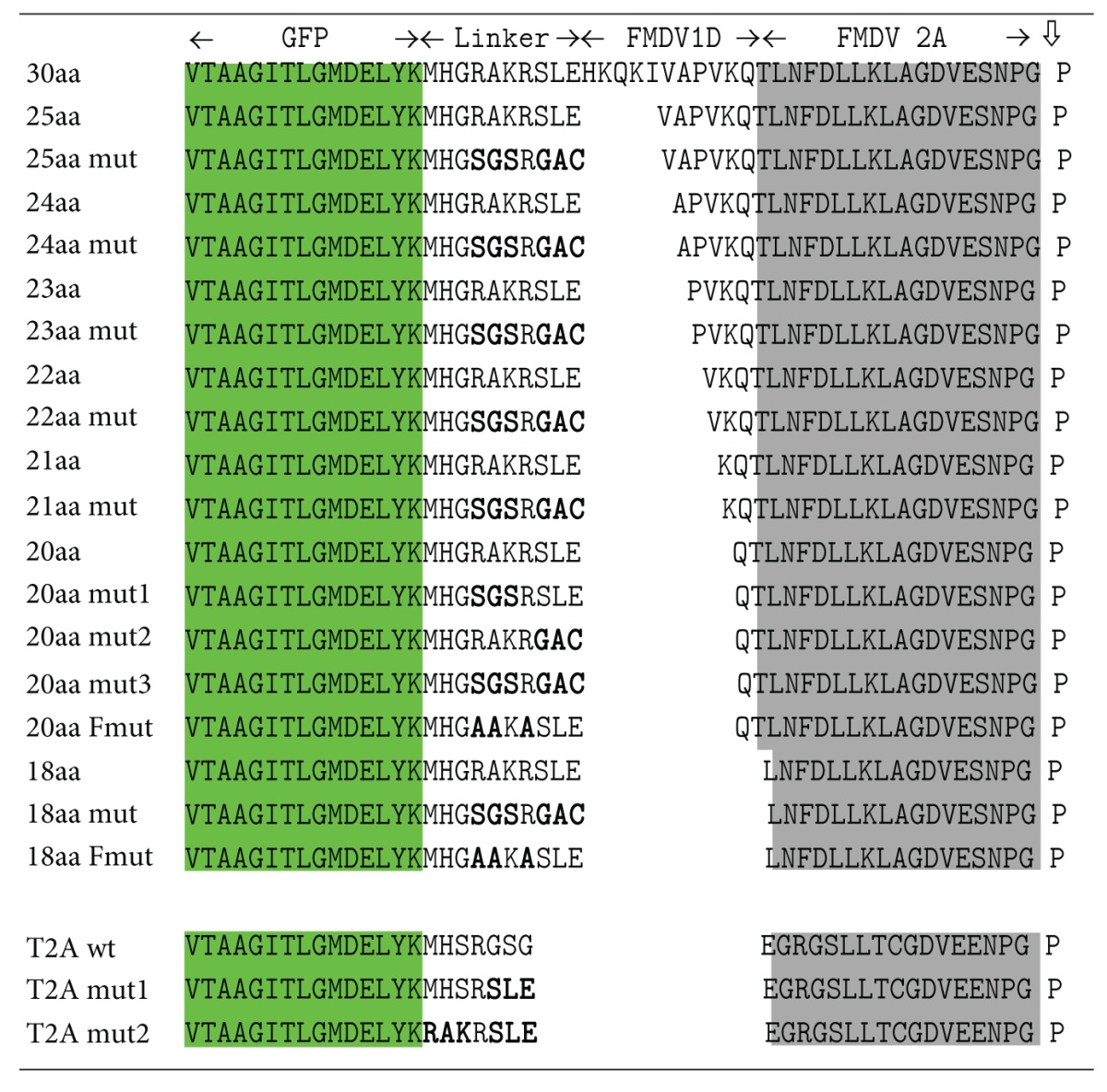# Protein Coexpression Using FMDV 2A: Effect of “Linker” Residues

**DOI:** 10.1155/2013/291730

**Published:** 2013-06-12

**Authors:** Ekaterina Minskaia, Martin D. Ryan

**Affiliations:** Biomedical Sciences Research Complex, North Haugh, University of St. Andrews, St. Andrews, Fife KY16 9ST, UK

## Abstract

Many biomedical applications absolutely require, or are substantially enhanced by, coexpression of multiple proteins from a single vector. Foot-and-mouth disease virus 2A (F2A) and “2A-like” sequences (e.g., *Thosea asigna* virus 2A; T2A) are used widely for this purpose since multiple proteins can be coexpressed by linking open reading frames (ORFs) to form a single cistron. The activity of F2A “cleavage” may, however, be compromised by both the use of shorter versions of F2A and the sequences (derived from multiple-purpose cloning sites) used to link F2A to the upstream protein. To characterise these effects, different lengths of F2A and T2A were inserted between green and cherry fluorescent proteins. Mutations were introduced in the linker region immediately upstream of both F2A- and T2A-based constructs and activities determined using both cell-free translation systems and transfected cells. In shorter versions of F2A, activity may be affected by both the C-terminal sequence of the protein upstream and, equally strikingly, the residues immediately upstream introduced during cloning. Mutations significantly improved activity for shorter versions of F2A but could decrease activity in the case of T2A. These data will aid the design of cloning strategies for the co-expression of multiple proteins in biomedical/biotechnological applications.

## 1. Introduction 

Many biomedical applications require vectors that can direct the expression of multiple proteins; subunits of hetero-multimeric proteins, multiple therapeutic genes (combined and/or synergistic effects), or, simply, coexpression of a therapeutic protein along with proteins that act as (selectable) markers of transformed cells [1, 2]. A number of approaches are used to coexpress multiple genes, including fusion proteins (which may include proteinase cleavage sites), alternative mRNA splicing, multiple promoters, reinitiation of translation, and internal ribosome entry sites (IRESes). Each, however, has associated disadvantages: fusion proteins localise to only a single subcellular site, while steric hindrance may alter their function. If a proteinase cleavage site is incorporated, this requires colocalisation of the substrate and processing enzyme in the same subcellular site. Internal promoters frequently show interference or are downregulated, while expression from IRESes (dependent on various cellular binding factors) varies between different cell types. Although derived from a single bicistronic mRNA, expression of the downstream ORF (IRES-driven cap-independent translation) is typically ~10% of that of the upstream ORF (cap-dependent translation). IRES elements, identified both in viral and cellular eukaryotic mRNAs, differ in nucleotide length (from 130 bp to 1 kb). However, the most efficient viral IRESes successfully utilized in vectors used for biomedical purposes are about 500 bp in length. Their comparatively large size can be a limiting factor when using virus-based vectors which have limited coding capacity: adeno-associated vectors cannot package more than ~5 kb efficiently, whilst retroviral vectors can package only ~7-8 kb [1–7].

Foot-and-mouth disease virus 2A (F2A) and “2A-like” sequences have become a useful alternative to these approaches since multiple proteins can be coexpressed at equimolar amounts from a single transcript mRNA under the control of a single promoter. 2A mediates a cotranslational “ribosome skipping” event (for simplicity referred to as “cleavage”), to produce the C-terminus of 2A. Interestingly, the length of 2A in the FMDV polyprotein (18aa) is defined by the site of the skipping event (forming the C-terminus of F2A), plus the N-terminus delineated by the site where a virus-encoded proteinase (3C^pro^) trims “2A” from the upstream capsid protein 1D at a later stage in virus replication. We have shown, however, that the functional length of “2A” actually incorporates (capsid protein 1D) sequences upstream of 2A. The longer versions of 2A described below are therefore 2A plus N-terminal extensions of the upstream capsid protein 1D, but for simplicity referred to as F2A [8–11].

The major advantages of using the 2A system in the construction of multicistronic vectors are (i) its small size (54–174 bp) compared to IRESes, (ii) that coexpression of proteins linked by 2A is independent of the cell type (since cleavage activity is only dependent on eukaryotic ribosomes, structurally highly conserved amongst the eukaryota), and (iii) that multiple 2As may be used, the activity of each being completely independent. In the case of F2A, it was demonstrated that although good cleavage was observed using the 19aa version, the use of longer versions of 2A was reported to produce higher levels of cleavage [8–11].

While the F2A sequence has been used widely, many “2A-like” sequences have been utilized successfully, including equine rhinitis A virus (E2A), porcine teschovirus-1 (P2A), and *Thosea asigna* virus (T2A) [9–17]. These “2A-like” sequences have been used in adoptive cell therapies [12, 14, 15], genetic engineering of human stem cells [16, 18, 19], and the coexpression of transcription factors in the induction of pluripotent stem cells [17, 20–23].

To date, F2A sequences of various lengths have been utilised in biotechnology, from 18 to 58aa [24–31]. In heterologous (artificial polyprotein) contexts, the 2A sequence remains as a C-terminal extension of the upstream gene: potentially a consideration when using longer, albeit more efficient, F2A sequences. C-terminal extensions may affect protein conformation, whilst an “authentic” C-terminus may be crucial for activity or for posttranslational modification. Shorter versions of 2A have been used, although often at the expense of cleavage efficiency [9, 12, 24, 27, 28]. In the case of proteins entering the exocytic pathway, C-terminal extensions of 2A may be “trimmed” away by the incorporation of a furin cleavage site between the upstream protein and 2A [24, 32]. Furthermore, it has been reported that in some cases the efficiency of 2A cleavage may be greatly inhibited when coexpressing certain proteins targeted to the exocytic pathway. C-terminal sequences of the upstream gene were implicated to be responsible for these lower levels of cleavage [33]. 

To further optimise the 2A coexpression system, we used T2A and shorter versions of F2A inserted between GFP and CherryFP fluorescent “reporter” proteins. Mutations were introduced into the region immediately upstream of both F2A- and T2A-based constructs. The effects on activity were determined using both cell-free coupled transcription/translation systems *in vitro* and in transfected HeLa cells, a human cell line most commonly used in biomedical applications. Activity can be affected in two ways: (i) by the C-terminal region of the protein upstream of F2A (where shorter F2As are utilised) and, significantly, (ii) by the short amino acid tract linking the protein upstream with F2A introduced by the cloning strategy. In the case of shorter versions of F2A, in some cases mutation significantly increased activity. Conversely, in the case of the highly active T2A, some mutations decreased activity. Taken together, these data show that sequences within 30–40aa upstream of the cleavage site may be highly influential in determining 2A activity: particular care should be taken in this aspect in the design of constructs for coexpression of multiple proteins prior to their further development in biomedical applications.

## 2. Materials and Methods

The following reagents were obtained from commercial suppliers: *Pfu* polymerase, T4 DNA ligase, restriction enzymes, JM109 competent cells, and Quick *TnT* rabbit reticulocyte lysate kit (Promega, Madison, WI, USA); ^35^S methionine (MP Biomedicals, Santa Ana, CA, USA); EDTA-free protease inhibitor cocktail (Sigma-Aldrich Company Ltd., Dorset, UK); pcDNA3.1, anti-RFP, rabbit purified polyclonal antibody against Red Fluorescent Protein (1 : 1000 dilution), anti-GFP, mAb 3E6, anti-Green Fluorescent Protein, mouse monoclonal antibody (1 : 1000 dilution), anti-*β*-tubulin, mouse monoclonal antibody (1 : 2000 dilution), Lipofectamine2000 transfection reagent, iBlot Gel Transfer Stacks, and nitrocellulose (Life Technologies Ltd., Paisley, UK); secondary HRP-conjugated polyclonal goat antimouse and antirabbit antibodies (Dako UK Ltd., Ely, UK); EZ-ECL Chemiluminescence Detection Kit for HRP (Geneflow Ltd., Elmhurst, UK); and Oligonucleotides were purchased from Integrated DNA Technologies (Coralville, IA, USA). 

### 2.1. Construction of Plasmids

*pGFP-F2As-CherryFP Plasmid Construct Series*. Here, F2As of various lengths (58, 30, 25–20, and 18aa) were inserted between sequences encoding GFP and CherryFP (Table 1). To make the pGFP-F2A_58_-CherryFP construct, the sequence encoding the 58aa version of F2A was obtained by digesting pSV7 plasmid (kind gift of Sandra Vater) with NsiI and ApaI. This insert was then used to replace the TaV 2A in pJC3, similarly restricted. Plasmid pGFP-F2A_58_-CherryFP was then used as a template to produce further truncated forms of F2A. Briefly, different lengths of F2A were amplified by PCR, creating an XhoI site at the N-terminus and a NotI site present downstream of the CherryFP in both plasmids. Forward oligonucleotides incorporating the XhoI restriction site (underlined)

 5′-GCGCTCGAGCACAAACAGAAAATTGTGGCACCGGTG-3′for F2A_30_,
 5′-GCGCTCGAGGTGGCACCGGTGAAACAGACTTTG-3′ for F2A_25_,
 5′-GCGCTCGAGGCACCGGTGAAACAGACTTTG-3′ for F2A_24_,
 5′-GCGCTCGAGCCGGTGAAACAGACTTTGAATTTTG-3′ for F2A_23_,
 5′-GCGCTCGAGGTGAAACAGACTTTGAATTTTGAC-3′ for F2A_22_,
 5′-GCGCTCGAGAAACAGACTTTGAATTTTGACCTTC-3′ for F2A_21_,
 5′-GCGCTCGAGCAGACTTTGAATTTTGACCTTCTCAAG-3′ for F2A_20_,
 5′-GCGCTCGAGTTGAATTTTGACCTTCTCAAGTTGGCG-3′ for F2A_18_ and a reverse oligonucleotide (5′-TAGAAGGCACAGTCGAGGC-3′, binds ~70 nts downstream of the NotI site) were used to amplify F2As-CherryFP with 2As of various lengths. The PCR products were digested with XhoI and NotI and cloned into pGFP-F2A_58_-CherryFP, similarly restricted.

*pGFP-F2As-CherryFP Mutant Constructs. *Truncated and mutated forms of F2A are shown in Table 1. These forms were produced by recombination in competent JM109 cells of two PCR products. The first was amplified with forward primer encoding the mutated sequences and a reverse primer binding to the vector backbone at nt 4500 (5′-GTTCCACTGAGCGTCAGACCCCGTAG-3′). The second was amplified using a reverse primer encoding the mutated sequence and a forward primer binding to the vector backbone at nt 4500 (5′-CTACGGGGTCTGACGCTC AGTGGAAC-3′). Forward and reverse primer sets for each construct are shown in Table  S1 (see Table  S1 in the species list in the Supplementary Material available online at http://dx.doi.org/10.1155/2013/291730).

### 2.2. Transcription and Translation

Plasmid constructs were used to programme Quick *TnT* transcription/translation reticulocyte lysate systems (Promega). Translation reactions (10 *μ*L) were performed according to the manufacturer's instructions. Briefly, 20 ng of plasmid DNA was used to programme rabbit reticulocyte lysates containing ^35^S methionine (10 *μ*Ci) and incubated at 30°C for 1 h. Translation reactions were stopped by the addition of 2x protein sample buffer. Protein products were analysed by 12% SDS-PAGE.

### 2.3. Expression in HeLa Cells

HeLa cells were maintained in DMEM medium supplemented with 10% FCS. The cells were transfected in 60 mm dishes (preplated 20 h earlier to 60% confluency) with 1.5 *μ*g of plasmid DNA and 7 *μ*L of Lipofectamine2000 (Invitrogen) in a final volume of 400 *μ*L OptiMEM. Transfection mix was added to 2 mL of antibiotic-free serum-containing medium and incubated for 5 h. Cells were further incubated for a total of 30 h after-transfection after addition of 2 mL of media containing 10% FCS. 

### 2.4. Western Blotting

30 h after-transfection, cells were washed twice with 1 mL of PBS and harvested in 1 mL of PBS by centrifugation at 2000 rpm for 5 min. Whole-cell lysates were prepared in 70 *μ*L of radioimmuno precipitation assay buffer, RIPA (150 mM NaCl, 10 mM Tris-HCl pH 7.4, 1% Triton X-100, 1% Na deoxycholate, 0.1% SDS), and freshly added 1/20 volume of EDTA-free protease inhibitor cocktail (Sigma) by incubation on ice for 30 min and centrifugation at 12000 rpm for 20 min at 4°C. Cellular debris was discarded and supernatants containing proteins were analysed by 12% SDS-PAGE. The proteins were then transferred to a nitrocellulose membrane, which was probed with anti-GFP or anti-CherryFP primary antibodies overnight, after blocking in PBST containing 5% milk for 1 h. Following overnight incubation, the membranes were washed three times in PBST. Detection of bound primary antibodies was performed with respective HRP-conjugated secondary antibodies (Dako), in PBST containing 5% milk for 1 h. The membranes were washed three times with PBST, rinsed in deionised water, and subjected to enhanced chemiluminescence by incubating the membranes in freshly prepared visualisation solution for 2 min. The membranes were exposed to an autoradiograph film for 3 to 45 seconds.

## 3. Results

### 3.1. Substitutions Immediately Upstream of F2A (20aa Version; F2A_20_): Effect on Cleavage Efficiency

**Figure 1:**
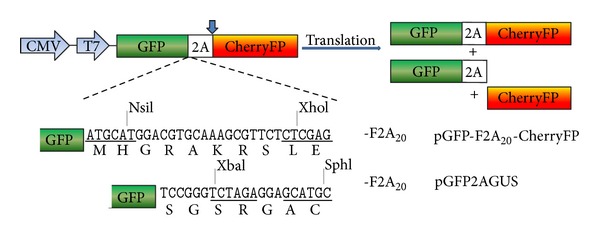
Sequence comparison between pGFP-F2A_20_-CherryFP and pGFP2AGUS constructs. While the downstream genes were different (CherryFP or GUS, resp.), both constructs had the same upstream GFP gene and F2A of 20aa. The only difference was in the sequence immediately upstream of F2A due to different restriction sites introduced for the specific cloning purposes.

**Figure 2:**
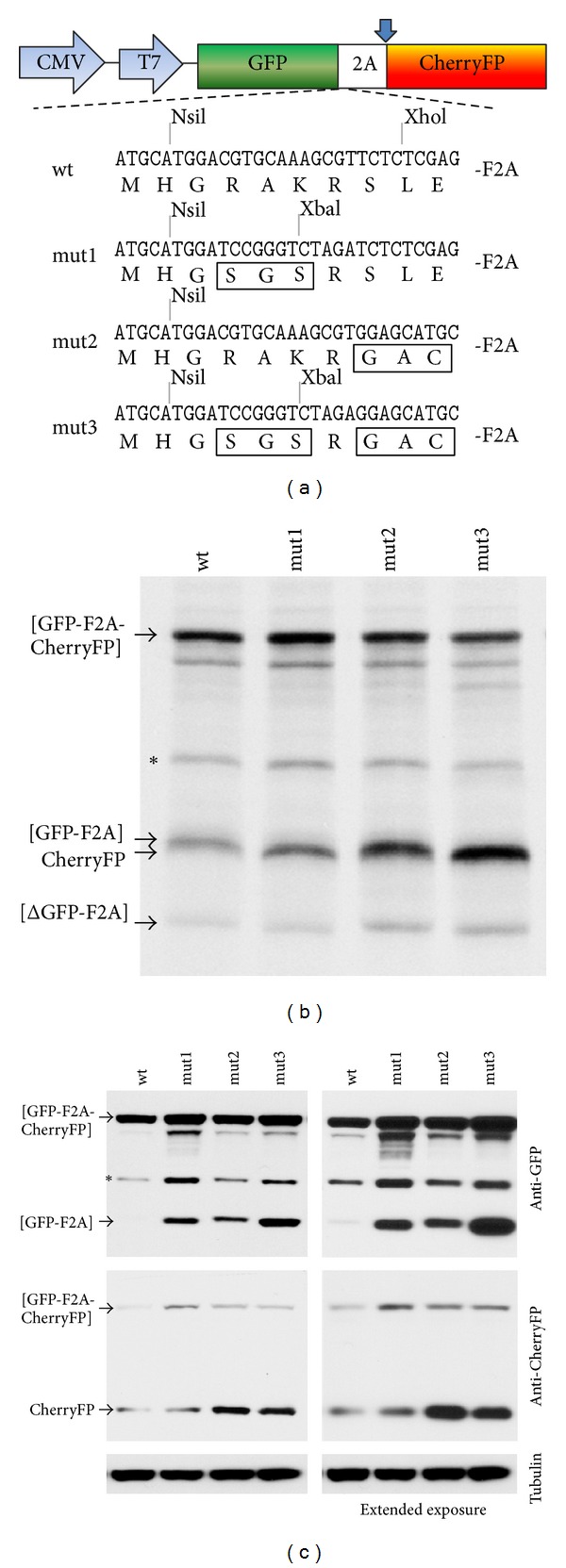
Effect of various substitutions, introduced in the region immediately upstream of F2A, on cleavage efficiency of [GFP-F2A_20_-CherryFP] polyproteins. (a) Amino acid sequence comparison (mutated residues are boxed) of parental pGFP-F2A_20_-Cherry (wt) and three mutant pGFP-F2A_20_-Cherry constructs (mut1, mut2, and mut3) used to coexpress GFP and CherryFP proteins from a single ORF *in vitro *using coupled transcription/translation rabbit reticulocyte lysate system (b) and transfected HeLa cells (c). For *TnT*, reticulocyte lysates were programmed with 20 ng of plasmid DNA, and translation products were resolved by the 12% SDS-PAGE. For *in vivo *studies, HeLa cells were transfected with 1.5 *μ*g of plasmid DNA and harvested 30 h after transfection. Cells were lysed in RIPA buffer, and equal amounts of total protein for each transfection were loaded onto 12% SDS-PAGE gel. The proteins were transferred onto a nitrocellulose membrane, blocked in PBS containing 5% milk and probed with anti-GFP (upper blot) and anti-CherryFP (middle blot) antibodies overnight at 4°C. Detection of bound primary antibody was achieved by using respective secondary antibodies, followed by ECL detection. All experiments were done in triplicate.

 Previously we showed that the 20aa version of F2A encoded by pGFP2AGUS was highly active [8, 27]. Indeed, this version has been used widely in other laboratories. To our surprise, the cleavage activity observed for the same length of F2A used to link GFP and CherryFP (pGFP-F2A_20_-CherryFP) was noticeably lower (Figures 1 and 2(c)). The upstream GFP and the F2A (20aa) were identical in both constructs: the only difference was the short “linker” between GFP and F2A created by the cloning strategy: -RAK**R**SLE- (pGFP-F2A_20_-CherryFP; linker derived from furin and XhoI restriction site) and -SGS**R**GAC- (pGFP2AGUS; linker derived from XbaI and SphI restriction sites), with only the arginine (shown in bold type face) in common (Figure 1). 

To investigate the reason underlying the different cleavage activities, we produced a set of three pGFP-F2A_20_-CherryFP mutant constructs in which the sequence of this linker was converted, stepwise (changed residues underlined), to the pGFP2AGUS linker sequence: mut1 (-RAKRSLE- substituted to -SGSRSLE-), mut2 (-RAKRSLE- to -RAKRGAC-), and mut3 (-RAKRSLE- to -SGSRGAC-) with the mut3 sequence being identical to that of the more active pGFP2AGUS sequence (Figures 1 and 2(a)).  Coupled transcription/translation analyses using *in vitro* systems produced five translation products. The major translation products corresponded to proteins of predicted molecular weights: uncleaved [GFP-F2A_20_-CherryFP] and the two cleavage products, [GFP-F2A_20_] and CherryFP, the latter two products comigrating on the gel due to similar mass (Figure 2(b)). The ratio of the cleaved : uncleaved products was higher in mutants 2 and 3, demonstrating higher cleavage activity in these constructs. Translation profiles derived from all four constructs also showed a certain level of internal initiation within GFP, producing the N-terminally truncated cleavage product [ΔGFP-F2A]. On the basis of the molecular mass of the product and distribution of methionine codons within GFP, we assume that this low-level internal initiation occurred at Met88—in a favourable Kozak context (cC**AUG**c). There was also a slower migrating band, corresponding to a protein of ~45 kDa, which was also present in western blots (Figure 2(c), indicated with an asterisk). This minor product was detected by anti-GFP (Figure 2(c), upper blot) but not anti-CherryFP antibodies (Figure 2(c), middle blot). The nature of this minor product is the subject of further characterisation. As expected, the upstream [GFP-F2A] cleavage product was detected by anti-GFP but not anti-CherryFP antibodies (Figure 2(c), upper blot), whilst the downstream CherryFP product was detected by anti-CherryFP but not anti-GFP, antibodies (Figure 2(c), middle blot). 

Data obtained from both *in vitro* translation and *in vivo* (transfected HeLa cells) using these four constructs showed that in the case of pGFP-F2A_20_-CherryFP, introduction of the -SGS- substitution (mut1) somewhat improved cleavage activity. Introduction of either the -GAC- substitution adjacent to the N-terminus of F2A (mut2) or the -SGSRGAC- substitution (mut3) resulted, however, in considerably higher cleavage activity, demonstrated by the increased ratio of cleaved : uncleaved products (more evident in western blots with extended exposures; Figures 2(b) and 2(c)). 

### 3.2. Substitutions Immediately Upstream of F2A (25–18aa Versions): Effect on Cleavage Efficiency

**Figure 3:**
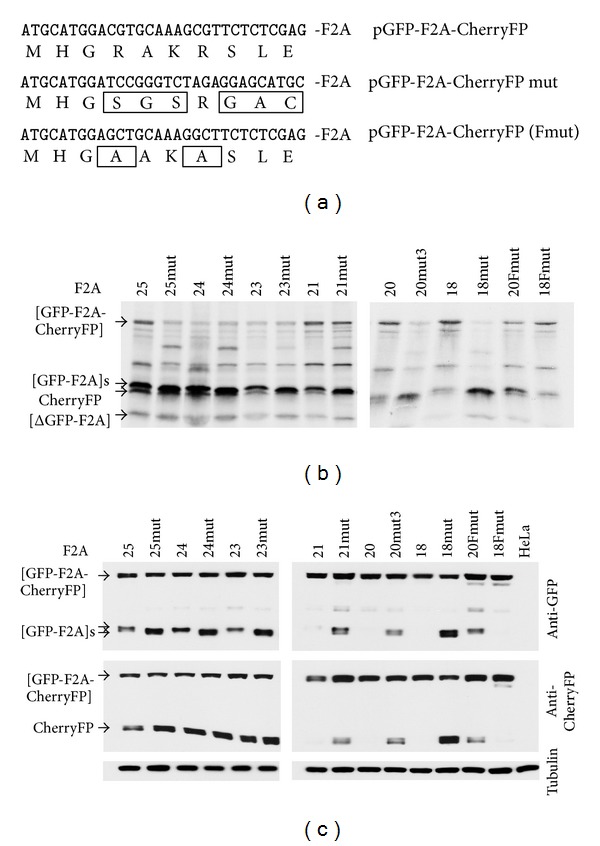
Effect of -RAKRSLE- to -SGSRGAC- substitution immediately upstream of F2A on cleavage efficiency of pGFP-F2A-Cherry constructs with F2As of 25–18aa. Parental pGFP-F2A-Cherry constructs and their mutant versions (mut or Fmut) (mutated residues are boxed, (a)) were used to coexpress GFP and CherryFP proteins from a single ORF *in vitro *using coupled transcription/translation rabbit reticulocyte lysate system (b) and transfected HeLa cells (c). For *TnT*, reticulocyte lysates were programmed with 20 ng of plasmid DNA, and translation products were resolved by the 12% SDS-PAGE. For *in vivo *studies, HeLa cells were transfected with 1.5 *μ*g of plasmid DNA and harvested 30 h after transfection. Cells were lysed in RIPA buffer, and equal amounts of total protein for each transfection were loaded onto 12% SDS-PAGE gel. The proteins were transferred onto a nitrocellulose membrane, blocked in PBS containing 5% milk, and probed with anti-GFP (upper blot) and anti-CherryFP (middle blot) antibodies overnight at 4°C. Detection of bound primary antibody was achieved by using respective secondary antibodies, followed by ECL detection. All experiments were done in triplicate.

**Figure 4:**
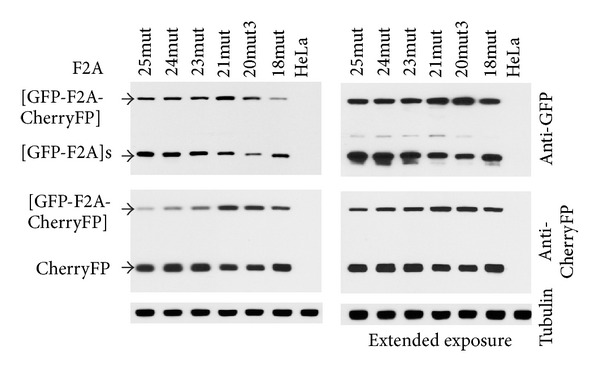
Efficiency of F2A cleavage in pGFP-F2A-CherryFP “mutants” with -SGSRGAC- substitution immediately upstream of F2A of 25–18aa. pGFP-F2A-CherryFP “mutant” constructs were used to coexpress GFP and CherryFP proteins from a single ORF in transfected HeLa cells. The cells were transfected with 1.5 *μ*g of plasmid DNA and harvested 30 h after transfection. Cells were lysed in RIPA buffer, and equal amounts of total protein for each transfection were loaded onto 12% SDS-PAGE gel. The proteins were transferred onto a nitrocellulose membrane, blocked in PBS containing 5% milk, and probed with anti-GFP (upper blot) and anti-CherryFP (middle blot) antibodies overnight at 4°C. Detection of bound primary antibody was achieved by using respective secondary antibodies, followed by ECL detection. All experiments were done in triplicate.

To investigate if such substitutions would affect the cleavage efficiency of constructs encoding longer versions of F2A (Table 1), we substituted the -RAKRSLE- linker with -SGSRGAC-. The effects of amino acid substitutions were determined using translation *in vitro* and in HeLa cells transfected with plasmid DNAs and western blotting (Figures 3(b) and 3(c)). The cleavage activity (ratio of cleaved : uncleaved forms) of all mutant constructs was higher (more apparent in western blots with extended exposures (see Figure  S1)). This difference was not as significant in longer versions (24/25aa) compared to shorter versions (18/20/21aa): the cleavage activity of the shortest version (18aa) was the most “improved.” Overall, the improved mutant constructs with resulting high cleavage efficiency followed the previously described “cleavage pattern” [27] and Minskaia et al., manuscript in preparation, where stepwise amino acid deletions from the N-terminus of F2A resulted in linear increase in accumulation of uncleaved polyprotein and a decrease in the amounts of cleaved products (Figure 4). This pattern, however, was changed in the case of the construct with the shortest version of F2A (pGFP-F2A_18mut_-CherryFP), as it was more efficient at cleavage, compared to pGFP-F2A_20mut_-CherryFP, judging by the decrease in the amount of uncleaved polyprotein and increase in the amounts of both [GFP-F2A] and CherryFP detected by the appropriate antibodies. Finally, we noticed that the cleaved [GFP-F2A] products of parental constructs migrated more slowly on the gel compared to those from the mutant constructs (Figure 3(c)). Substitution of the -SGSRGAC- (637 Da) by -RAKRSLE- (859 Da) decreased the molecular weight of mutant cleavage products by more than 200 Da. 

In order to more finely map the effect of these mutations, the -RAKR- motif within the 18 and 20aa versions of pGFP-F2A-CherryFP (encoding the least active F2As) was mutated to -AAKA-, producing pGFP-F2A_20Fm_-CherryFP and pGFP-F2A_18Fm_-CherryFP (Figures 3(a) and Table 1). Interestingly, the cleavage activity for the pGFP-F2A_18Fm_-CherryFP construct was as low as that of the parental pGFP-F2A_18_-CherryFP construct, demonstrating that the improved cleavage efficiency of pGFP-F2A_18mut_-CherryFP with -SGSRGAC- substitution was, therefore, attributable to the nature of the linker sequence (Figures 3(c) and Table 1). On the contrary, introduction of the -AAKA- substitution in the F2A_20_ construct resulted in an increase in cleavage efficiency comparable to that of the mutant construct pGFP-F2A_20mut_-CherryFP with the -SGSRGAC- substitution. These results demonstrate that the difference in the 2 residues at the N-terminus of F2A (pGFP-F2A_20Fm_-CherryFP *versus* pGFP-F2A_18Fm_-CherryFP), together with the difference in the 3 residues immediately upstream of F2A (-SGSR**GAC**- *versus* -AAKA**SLE**- in the F2A_18_ construct), can have a profound effect on cleavage efficiency in the case of these short F2As (Figures 3(c) and Table 1). 

### 3.3. Introduction of Mutations Immediately Upstream of T2A May Decrease Activity

**Figure 5:**
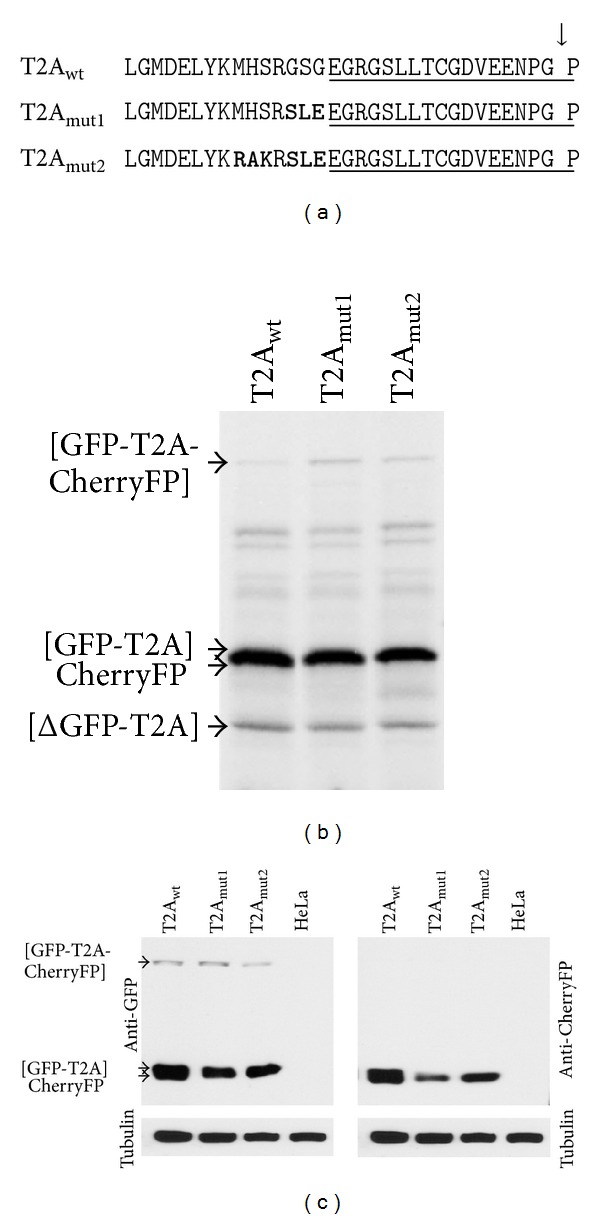
Effect of “reverse” substitutions, immediately upstream of T2A, on cleavage efficiency of pGFP-T2A-CherryFP constructs. (a) Amino acid substitutions introduced in pGFP-T2A-CherryFP sequence immediately upstream of T2A. Mutated residues are shown in bold, and T2A sequence is underlined. Parental construct pGFP-T2A-CherryFP with -MHSRGSG- linker (wt) and its mutant versions with -MHSRSLE- and -RAKRSLE- substitutions (T2A_mut1_ and T2A_mut2_, resp.) were used to coexpress GFP and CherryFP proteins from a single ORF *in vitro *using coupled transcription/translation rabbit reticulocyte lysate system (b) and in transfected HeLa cells (c). For *TnT*, reticulocyte lysates were programmed with 20 ng of plasmid DNA, and translation products were resolved by the 12% SDS-PAGE. For *in vivo *studies, HeLa cells were transfected with 1.5 *μ*g of plasmid DNA and harvested 30 h after transfection. Cells were lysed in RIPA buffer, and equal amounts of total protein for each transfection were loaded onto 12% SDS-PAGE gel. The proteins were transferred onto a nitrocellulose membrane, blocked in PBS containing 5% milk, and probed with anti-GFP (left blot) and anti-CherryFP (right blot) antibodies overnight at 4°C. Detection of bound primary antibody was achieved by using respective secondary antibodies, followed by ECL detection. All experiments were done in triplicate.

The T2A sequence (18aa) is highly active and has not been reported to be susceptible to the nature of the C-terminus of the upstream gene. To test if T2A was susceptible to substitutions immediately upstream, we introduced “reverse” substitutions (mut1: MHSRGSG- to -MHSRSLE- and mut2: -MHSRGSG to -RAKRSLE-) in the same [GFP-2A-CherryFP] context (Figures 5(a) and Table 1). The effect on cleavage activity was determined using reticulocyte lysate *in vitro* translation systems (Figure 5(b)) and transfected HeLa cells (Figure 5(c)). These substitutions resulted in decreased cleavage activity in both mutant constructs, more apparent in HeLa cells than in reticulocyte lysates, as detected by both anti-GFP (Figure 5(c), left blot) and anti-CherryFP (Figure 5(c), right blot) antibodies. Introduction of the -SLE- substitution immediately upstream of T2A (mut1; Table 1) had even greater effect on activity than the -RAKRSLE- substitution (mut2; Table 1; Figure 5(c)). Again, this observation reveals the importance of the 3 residues immediately upstream of such short F2As. It is worth noting that in our parental construct, the -MHSRGSG- sequence comprises 3 restriction sites used for cloning (NsiI, XbaI, and BspEI). Coincidentally, a -GSG- linker motif was recently shown to improve cleavage efficiency ([12, 34], discussed below). 

### 3.4. Comparison of Cleavage Efficiencies of “Improved” Short F2As and T2A

**Figure 6:**
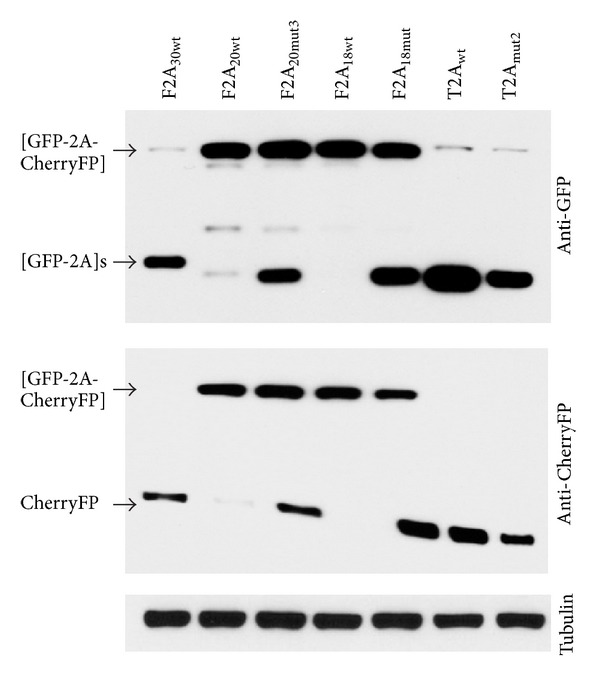
Efficiency of cleavage in constructs with short 2A sequences. pGFP-2A-CherryFP constructs with F2As 30_wt_, 20_wt_, 20_mut3_, 18_wt_, 18_mut_, and T2A_wt_ and T2A_mut2_ were used to coexpress GFP and CherryFP proteins from a single ORF in transfected HeLa cells. The cells were transfected with 1.5 *μ*g of plasmid DNA, and harvested 30 h after transfection. Cells were lysed in RIPA buffer, and equal amounts of total protein for each transfection were loaded onto 12% SDS-PAGE gel. The proteins were transferred onto a nitrocellulose membrane, blocked in PBS containing 5% milk, and probed with anti-GFP (upper blot) and anti-Cherry (middle blot) antibodies overnight at 4°C. Detection of bound primary antibody was achieved by using respective secondary antibodies, followed by ECL detection. All experiments were done in triplicate.

As a last step in optimisation of the F2A system, we compared cleavage efficiencies of the most active F2A- and T2A-based constructs (F2A_30_, F2A_20mut3_, F2A_18mut_, and T2A_wt_) in HeLa cells (Figure 6). Two major products corresponding to proteins of expected molecular weights, uncleaved [GFP-2A-CherryFP] polyprotein and the two cleavage products, [GFP-2A] and CherryFP, were detected by western blot analysis with both anti-GFP (Figure 6, upper blot) and anti-CherryFP (Figure 6, middle blot) antibodies. The cleavage efficiency of the F2A_30_ version, shown to be highly efficient F2A (Minskaia et al., manuscript in preparation), was comparable to that of the T2A_wt_- and T2A_mut2_-versions, with virtually no uncleaved polyprotein present in HeLa cells. Substitutions immediately upstream of T2A (T2A_mut2_) resulted in decreased coordinate expression of both genes. This construct was still highly active compared to F2A_20wt_- and F2A_18wt_-based versions with identical amino acids immediately upstream of F2A. Their substitution to the more efficient F2A_20mut3_- and F2A_18mut_-based constructs resulted in significant increase in the proportion of cleaved products. Some uncleaved product was, however, observed in HeLa cells transfected with these constructs. 

## 4. Discussion

The model for 2A-mediated cleavage predicts that the nascent 2A interacts with the exit tunnel of the ribosome to affect the conformational space occupied by the ester bond linking the nascent protein and tRNA^gly^ in the ribosome P-site. The model predicts, therefore, that residues that may influence activity reside within the exit tunnel of the ribosome [35–37]. Ours and others, data indicate that in the case of F2A this interaction with the tunnel occurs along the entire length of F2A, whereas in the case of T2A it appears that the net effect of this tunnel interaction is accomplished by a shorter tract. This notion is supported by our recent data (Minskaia et al., manuscript in preparation) in which we show that the cleavage activity of F2As of 30aa (and longer) was insensitive to the C-terminal sequence of the upstream gene. Stepwise N-terminal amino acid deletions resulted, however, in dramatically decreased cleavage efficiencies. Taken together, these observations were consistent with structural data showing that the ribosome exit tunnel may accommodate 30–40 amino acids [38]. The activity of shorter (25–18aa) forms of F2A may, therefore, be affected by the nature of the C-terminal sequence of the protein upstream since this region would now lie within the exit tunnel of the ribosome and affect its interaction with F2A. 

The aim of this study was to further characterise and improve the efficiency of the 2A coexpression system by introducing various mutations immediately upstream of shorter versions of F2A and T2A linking GFP and CherryFP proteins. Based on the results obtained *in vitro* in reticulocyte lysates and in HeLa cells transfected with plasmid DNAs, we demonstrate that introduction of the -SGS- substitution (mut1) 4 amino acids upstream of 20aa—long F2A slightly improved cleavage efficiency, while introduction of either -GAC- substitution adjacent to the N-terminus of F2A (mut2, Table 1) or -SGSRGAC- substitution (mut3, Table 1) immediately upstream of F2A resulted in considerably higher cleavage efficiencies (Figures 2(b) and 2(c)). 

**Figure 7:**
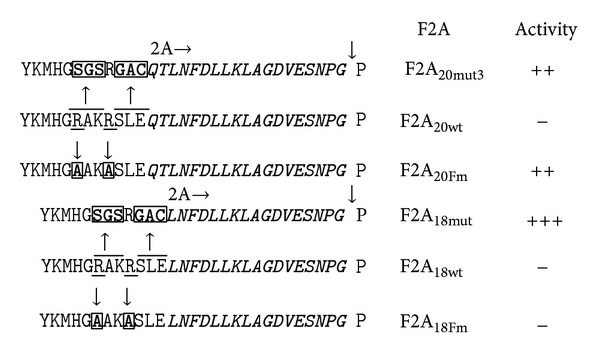
Location of key substitutions that affect 2A cleavage activity. Amino acid sequence comparison of parental pGFP-F2A_20_-CherryFP (F2A_20wt_) and pGFP-F2A_18_-CherryFP (F2A_18wt_) and their corresponding mutant derivatives (F2A_20mut_ and F2A_20Fm_, F2A_18mut_, and F2A_18Fm_) used to coexpress GFP and CherryFP proteins from a single ORF (F2A sequence in italics). Mutated residues are boxed, and cleavage activities are indicated by “−” or “+”.

We further demonstrate that in the case of the shortest F2As (21/20/18aa), substitution of the parental -RAKRSLE- sequence with -SGSRGAC- greatly improved cleavage efficiencies (Figures 3(a)–3(c), 4, and Table 1), an effect somewhat less evident in the case of longer F2As (25/23aa). The 18aa version, which showed relatively low activity, was dramatically improved by the introduction of these mutations. This increase in activity may be attributed to the -SLE- to -GAC- exchange since the (furin linker) -RAKR- to -AAKA- exchange had no detectable effect. However, in the case of the 20aa version, identical substitutions within either portion of the linker (-RAKR/SLE-) only slightly increased activity (Figure 3(c)). Introduction of either -SGSRGAC- or -AAKASLE- substitutions in the 20aa F2A version resulted in comparable increase in the cleavage activity. These results show that just 2aa at the N-terminus of “suboptimal” 2As (F2A_20_, F2A_18_), and the 3 residues immediately upstream of 2A (F2A_18mut_ *versus* F2A_18Fm_), can have a profound effect on F2A cleavage efficiency (Figure 7). We further showed that introduction of the “reverse” substitutions of -MHSRGSG- to -MHSRSLE- (mut1, Table 1) and to -RAKRSLE- (mut2, Table 1) in the highly active T2A-based construct resulted in *decreased* cleavage activity in both mutant constructs (Figures 5(a)–5(c)). Again, these data highlight the importance of the 3aa immediately upstream of T2A (in this instance). Finally, we show that cleavage efficiencies of F2A_30_-, T2A_wt_-, and T2A_mut2_-based constructs were similar, with virtually no uncleaved form present in HeLa cells (Figure 6). While introduction of the “reverse” substitutions upstream of T2A resulted in decreased coordinate expression of both genes (T2A_mut2_), it was still highly active compared to F2A_20wt_ and F2A_18wt_ with identical upstream residues. Their substitution to more “optimal,” F2A_20mut3_ and F2A_18mut_, resulted in significant increase in the amounts of cleaved products. The presence of some uncleaved translation product does not, however, compare favourably with the “high-cleavers” F2A_30wt_ and T2A_wt_. 

The data presented here is in agreement with previously published studies that successfully used F2A sequences of various lengths in *in vitro* and *in vivo* heterologous systems [24, 25, 27–29, 31]. To avoid using longer F2A sequences that remain attached at the C-terminus of the upstream protein, researchers have used shorter F2As but have reported a range of cleavage activities for F2As of the same length [9, 12, 24, 27, 28, 39]. Klump and colleagues [40] coexpressed the homeobox transcription factor along with GFP [GFP-F2A_24_-HoxB4]. The 24aa version of F2A produced small amounts of uncleaved translation product in cells—similar to the data we present here. Based on this work, Milsom and colleagues [41] first tested two constructs, pHoxB4-2A-GFP and pMGMT-2A-GFP, coexpressing HoxB4 and MGMT (O^6^-methylguanine-DNA-methyl-transferase, a transgene that conveys chemoresistance) with GFP and noted that in cells transfected with these constructs the amount of uncleaved [MGMT-F2A_24_-GFP] polyprotein was far greater than that of [HoxB4-F2A_24_-GFP]. Noting that the C-terminal sequence of the upstream protein could be the reason for decreased cleavage efficiency, a tricistronic retroviral vector pHoxB4-F2A_24_-MGMT-IRES-GFP was developed to simultaneously coexpress the three proteins in transfected bone marrow. With HoxB4 as the upstream gene, this tricistronic vector was concluded to be the best for coexpression of both genes, with insignificant amount of uncleaved polyprotein [41]. Similarly, using a 24aa version of F2A, de Felipe and Ryan [39] produced a series of constructs for coexpression of various fluorescent proteins (some including internal cotranslational signal sequences). Uncleaved polyprotein was detected, particularly for constructs encoding proteins targeted to the exocytic pathway [39]. This lowering of activity was attributed to interaction of the nascent peptide with the translocon pore (Sec61 complex) also affecting the interaction of F2A with the ribosome exit tunnel [33]. In the case of proteins targeted to enter the exocytic pathway, successful utilization of a 24aa version F2A with a furin cleavage site immediately upstream was reported by several groups [24, 32]. 

 This approach was used to link the antibody heavy and light chain sequences to engineer a mAb expression cassette that, in the context of AAV-mediated gene transfer, resulted in high levels of full-length, functional monoclonal antibodies *in vitro* and *in vivo *[28]. Using the same strategy, significant antitumour responses have been observed in the clinic using monoclonal antibodies that block immune checkpoints by coexpressing CTLA-4 heavy and light chains [42], while Camper and colleagues [32] designed a retroviral vector for the expression of a target Fab' fragment as a single polyprotein and showed equimolar expression and processing of the light and truncated heavy chain domains [32].

As for shorter F2As, different cleavage activities (depending on protein arrangement) were observed when GFP, EGFP, and hrGFP were coexpressed with cytochrome P450 2B1 (CYP2B1) using a 20aa version of F2A [43]. With a 19aa version of F2A, cleavage was reported to be less efficient (70%) for [CFP-F2A_19_-PAC], with some uncleaved polyprotein still present [44]. Similar results were achieved when an 18aa version of F2A was used for the generation of retroviral vectors [45]. Ma and Mitra [46] used even shorter F2A of 17aa to coexpress GUS and CAT proteins (in alternative gene orders) *in planta* and demonstrated that [GUS-F2A_17_-CAT] and [GUS-F2A_17_-GUS] were cleaved to completion, while [CAT-F2A_17_-GUS] was not. They concluded that the difference in cleavage efficiency was due to different upstream genes. In light of our experiments, however, it is noteworthy to mention that, based on the data provided, these constructs also differed in the sequence immediately upstream of 2A: -QQGGKQVD- for GUS-F2A_17_ and -EWQGGAVD- for CAT-F2A_17_ [46]. 

Interestingly, cleavage efficiency of shorter F2As in different contexts was shown to be improved by insertion of various spacer sequences, such as -SGS- or -GSG- [12, 34, 47, 48], the V5 epitope tag (-GKPUPNPLLGLDST-) [24], or a 3xFlag epitope tag [31] immediately upstream of F2A. It has been reported that in *γ*-retroviral constructs, expression of multiple genes linked with F2A peptides was facilitated by a spacer sequence (-SGSG- or -GSG-) immediately upstream of F2A_22_ [12, 34], while Yang and colleagues [24] tested both these spacers plus the V5 tag which acted, in this context, as a spacer. In these studies, lentiviral vectors expressing two-gene T-cell receptors directed against the melanoma differentiation antigens gp100 and MART-1 were constructed using F2A_24_. The addition of spacer sequences was, however, shown to be a prerequisite for efficient synthesis and assembly of biologically active T-cell receptor complexes. Interestingly, when the spacer sequence in the form of V5 tag was inserted between furin and F2A_24_, this increased activity and yielded optimal TCR expression in transduced lymphocytes. At the same time, attempts to substitute the V5 peptide with other sequences were unsuccessful, suggesting that the specific amino acid sequence of the V5 tag was responsible for enhanced ribosomal skipping in this context [24]. Recently, Tan and colleagues [31] successfully used a similar strategy by providing a spacer in the form of 3xFlag tag repeats (incorporated for other purposes) upstream of an 18aa version of F2A to express double or triple copies of the rabies virus glycoprotein from a single ORF in human adenovirus 5 (linked *via* F2As and followed by hrGFP as a marker after IRES).

Comparing the activity of 2As of different origins, Donnelly and colleagues [27] showed that T2A_20_ has the highest cleavage efficiency followed by E2A_20_, P2A_19_, and F2A_20_, while Szymczak and colleagues [12] demonstrated that F2A_22_ (-GSG- or -SGSG- upstream) and T2A_18_ have higher activities than E2A_20_. Funston and colleagues [29] reported that in the case of protein IX, essential for the packaging of full-length adenoviral genomes and selected as a fusion partner for coexpression of foreign genes in adenoviruses, [IX-F2A_58_-GFP] showed a higher activity than [IX-P2A_22_-GFP]. E2A, T2A, and P2A were successfully used to generate iPS cells from somatic cells by simultaneous lentiviral vector transduction of four transcription factors in a [KLF4-E2A_20_-OCT3/4-T2A_18_-SOX2-P2A_19_-c-Myc]-IRES-hrGFP construct [20]. Recently, Kim and colleagues [49] evaluated cleavage efficiency of P2A_19_, T2A_18_, E2A_20_, and F2A_22_ preceded by a -GSG- spacer in a [GFP-2A-CherryFP] context in three commonly used cell lines, zebrafish embryos, and adult mice, and concluded that P2A_19_ had the highest cleavage efficiency followed by T2A_18_, E2A_20_, and F2A_22_. 

## 5. Conclusions 

2A sequences have enabled more complex strategies of coexpression and transgenesis and have been used in many laboratories with highly successful results. Based on the data presented here and from previous studies, the variations in cleavage activity of the various lengths of F2As are attributed to the nature of the region immediately upstream of F2A (18–30aa upstream of the cleavage site), altered by residues introduced by the cloning strategy, or, by the introduction of spacer sequences. Indeed, in the case of the highly active T2A, changes in this region can result in a *decrease* of cleavage efficiency. Such variations in cleavage activity can, however, be manipulated to great purpose to produce “molecular rheostats.” 

In a very elegant series of experiments, Yu and colleagues produced a range of truncated and point-mutated forms of F2A and I2A (from the insect infectious flacherie virus) to regulate the ratio of surface-anchored to secreted forms of the immunoglobulin IgG [50]. The data presented in this paper (together with others), therefore, aids the design (and troubleshooting) of coexpression strategies but also serves to show how manipulation of these sequences can be used in expanding the range of such molecular rheostats.

## Supplementary Material

Supplementary Material Table S1. Primer sequences used for introduction of mutations in parental constructs. For each mutant (1D2A25-18mut, 1D2A20Fm, 1D2A18Fm, TaV2Amut1 and mut2) two PCR products were recombined in competent JM109 cells. The first was amplified with forward primer (Fw) encoding the mutated sequence (shown in lowercase) and a reverse primer binding to the vector backbone at nt 4500 (5'-GTTCCACTGAGCGTCAGACCCCGTAG-3'). The second was amplified using a reverse primer (Rev) encoding the mutated sequence (shown in lower case) and a forward primer binding to the vector backbone at nt 4500 (5'-CTACGGGGTCTGACGCTCAGTGGAAC-3').

## Figures and Tables

**Table 1:** Upstream, linker and F2A and T2A sequences in pGFP-2A-Cherry constructs.