# Supplementary material for: Protein Coexpression Using FMDV 2A: Effect of “Linker” Residues
Source: Biomed Res Int. 2013 Jun 12;2013:291730. doi: 10.1155/2013/291730 (PMC3710640; doi:10.1155/2013/291730)
Supplement: Supplementary file 1 — Supplementary Material Table S1. Primer sequences used for introduction of mutations in parental constructs. For each mutant (1D2A25-18mut, 1D2A20Fm, 1D2A18Fm, TaV2Amut1 and mut2) two PCR products were recombined in competent JM109 cells. The first was amplified with forward primer (Fw) encoding the mutated sequence (shown in lowercase) and a reverse primer binding to the vector backbone at nt 4500 (5'-GTTCCACTGAGCGTCAGACCCCGTAG-3'). The second was amplified using a reverse primer (Rev) encoding the mutated sequence (shown in lower case) and a forward primer binding to the vector backbone at nt 4500 (5'-CTACGGGGTCTGACGCTCAGTGGAAC-3'). [file 291730.f1.docx]

| F2A_25mut_  Fw  Rev  F2A_24mut_  Fw  Rev  F2A_23mut_  Fw  Rev  F2A_22mut_  Fw  Rev  F2A_21mut_  Fw  Rev  F2A_20mut1_  Fw  Rev  F2A_20mut2_  Fw  Rev  F2A_20mut3_  Fw  Rev  F2A_20Fm_  Fw  Rev  F2A_18mut_  Fw  Rev  F2A_18Fm_  Fw  Rev  T2A_mut1_  Fw  Rev  T2A_mut2_  Fw  Rev | GAGCTGTATAAGATGCATGGAtccgggtctagaggagcatgcGTGGCACCGGTGAAACAGACTTTGAATTTTGCAAAATTCAAAGTCTGTTTCACCGGTGCCACgcatgctcctctagacccggaTCCATGCATCTTATACAGCTC  GAGCTGTATAAGATGCATGGAtccgggtctagaggagcatgcGCACCGGTGAAACAGACTTTGAATTTTGAC  GTCAAAATTCAAAGTCTGTTTCACCGGTGCgcatgctcctctagacccggaTCCATGCATCTTATACAGCTC  GAGCTGTATAAGATGCATGGAtccgggtctagaggagcatgcCCGGTGAAACAGACTTTGAATTTTGAC GTCAAAATTCAAAGTCTGTTTCACCGGgcatgctcctctagacccggaTCCATGCATCTTATACAGCTC  GAGCTGTATAAGATGCATGGAtccgggtctagaggagcatgcGTGAAACAGACTTTGAATTTTGACCTTC  GAAGGTCAAAATTCAAAGTCTGTTTCACgcatgctcctctagacccggaTCCATGCATCTTATACAGCTC  GAGCTGTATAAGATGCATGGAtccgggtctagaggagcatgcAAACAGACTTTGAATTTTGACCTTCTC  GAGAAGGTCAAAATTCAAAGTCTGTTTgcatgctcctctagacccggaTCCATGCATCTTATACAGCTC  GAGCTGTATAAGATGCATGGAtccgggtctagaTCTCTCGAGCAGACTTTGAATTTTG  CAAAATTCAAAGTCTGCTCGAGAGAtctagacccggaTCCATGCATCTTATACAGCTC  ATGCATGGACGTGCAAAGCGTggagcatgcCAGACTTTGAATTTTGACCTTCTC  GAGAAGGTCAAAATTCAAAGTCTGgcatgctccACGCTTTGCACGTCCATGCATC  GAGCTGTATAAGATGCATGGAtccgggtctagaggagcatgcCAGACTTTGAATTTTGACCTTCTC  GAGAAGGTCAAAATTCAAAGTCTGgcatgctcctctagacccggaTCCATGCATCTTATACAGCTC  CGAGCTGTATAAGATGCATGGAgctgcaaaggctTCTCTCGAGCAGACTTTGAATTTTGAC  CAAAATTCAAAGTCTGCTCGAGAGAagcctttgcagcTCCATGCATCTTATAC  GAGCTGTATAAGATGCATGGAtccgggtctagaggagcatgcTTGAATTTTGACCTTCTCAAGTTGGCG  CGCCAACTTGAGAAGGTCAAAATTCAAgcatgctcctctagacccggaTCCATGCATCTTATACAGCTC    CGAGCTGTATAAGATGCATGGAgctgcaaaggctTCTCTCGAGTTGAATTTTGACCTTCTC  GAAGGTCAAAATTCAACTCGAGAGAagcctttgcagcTCCATGCATCTTATAC  CTCGGCATGGACGAGCTGTATAAGATGCATTCTAGAagcttagagGAGGGCAGGGGAAGTCTTCTAACATCATGTTAGAAGACTTCCCCTGCCCTCctctaagctTCTAGAATGCATCTTATACAGCTCGTCCATGCCGAG  CTCGGCATGGACGAGCTGTATAAGagggctaagAGAagcttagagGAGGGCAGGGGAAGTCTTCTAACATG  CATGTTAGAAGACTTCCCCTGCCCTCctctaagctTCTcttagccctCTTATACAGCTCGTCCATGCCGAG |
| --- | --- |

**Table S1. Primer sequences used for introduction of amino acid substitutions in parental pGFP-2A-CherryFP constructs.** Mutated nucleotides are shown in lowercase
